# Parathyroid Hormone as a Novel Biomarker for Chronic Obstructive Pulmonary Disease: Korean National Health and Nutrition Examination Survey

**DOI:** 10.1371/journal.pone.0138482

**Published:** 2015-09-23

**Authors:** Joo-Hyun Park, Hye Kyeong Park, Hoon Jung, Sung-Soon Lee, Hyeon-Kyoung Koo

**Affiliations:** Division of Pulmonary and Critical Care Medicine, Department of Internal Medicine, Ilsan Paik Hospital, Inje University college of Medicine, Goyang, Korea; University of Palermo, ITALY

## Abstract

**Objective:**

To understand and predict chronic obstructive pulmonary disease (COPD), a biomarker that reflects disease severity is needed.

**Research Design and Methods:**

Data from 10269 adults aged over 40 years of age were retrieved from the Korea National Health and Nutrition Examination Survey (KNHANES), and 1302 patients met the criteria for COPD. The association between values of vitamin D and parathyroid hormone (PTH), and COPD severity including lung function and quality of life, were analyzed.

**Results:**

In COPD patients, lung function was inversely related to PTH values (*P* = 0.02 for FVC [% predicted]; *P* < 0.001 for FEV_1_ [% predicted]); however, the association of lung function with vitamin D levels was not statistically significant in a multivariable analysis. Value of PTH was independently associated with EQ5D-index (*P* = 0.04), but vitamin D level showed no significant relationship with EQ5D-index (*P* = 0.59) or EQ5D-VAS (*P* = 0.81).

**Conclusions:**

Elevation of PTH, unlike vitamin D, is independently associated with COPD severity, and may be a better biomarker for COPD.

## Introduction

Chronic obstructive pulmonary disease (COPD) is a complex, heterogeneous disease [1]. To understand and predict these complexities, there has been great efforts to identify biomarkers that correlate with disease severity. One of the proposed biomarker for COPD is plasma vitamin D level. However, previous studies have shown inconsistent results regarding the role of vitamin D in lung function [2], COPD development [3, 4], exacerbation [5–7], comorbidities [8], and mortality [7]. Vitamin D is synthesized in the skin after sunlight exposure, but level of vitamin D can be influenced by food intake and several regulatory mechanisms, such as parathyroid hormone (PTH). The purpose of our study is to identify the biomarkers that reflect disease severity of COPD including lung function and quality of life by using data from the Korea National Health and Nutrition Examination Survey (KNHANES).

## Material and Methods

### Study design and participants

This study used data from KNHANES, a cross-sectional, nationally representative population-based health and nutritional surveys by the Korean Centers for Disease Control and Prevention since 1998. Participants were selected using proportional allocation systematic sampling with multistage stratification, and the sampling units were based on geographical area, age, and sex. This survey includes a health interview, a health examination, and nutritional questionnaires. Pulmonary function tests (PFTs) were performed for subjects >40 years of age. COPD was defined as a PFT result of FEV_1_/FVC <0.7 among adults aged over 40 years of age according to the Global Initiative for Chronic Obstructive Lung Disease (GOLD) guidelines [1]. Data from a total of 10269 adults >40 years of age were retrieved from KNHANES, and among them, population of 1302 patients who met the definition of COPD were included for further analysis. All the individuals in this surveillance participated voluntarily, and provided written informed consent. The survey protocol was approved by the institutional review board of the Korean Centers for Disease Control and Prevention.

### Measurements of variables

Spirometry was performed using standardized equipment (model 1022 Spirometer; SensorMedics, California, USA) according to guidelines from the American Thoracic Society/European Respiratory Society [9]. Spirometry was repeated at least three times to ensure validity and reproducibility. The PFT results were analyzed based on the reference values from predictive equations for Korean populations [10], and reviewed by trained physicians. As this study was part of a national survey, post-bronchodilator testing was not performed, and all measurements were based on pre-bronchodilator values. Blood samples were collected in serum separator tubes. Plasma was separated by centrifugation and stored at -70°C. Total serum 25-hydroxyvitamin D (25-OHD) level was measured using radioimmunoassay kits (Diasorin, Stillwater, MN, USA), and expressed in ng/mL (conversion factor, 2.5 for nmol/L). Plasma PTH was measured by N-tact PTH assay using the LIAISON (Diasorin, Stillwater, MN, USA) chemiluminescence immunoassay method, and expressed in pg/mL. Other parameters such as white blood cell (WBC) count, hemoglobin (Hb), platelets, blood urea nitrogen (BUN), and creatinine (Cr) were analyzed in a central, certified laboratory.

Questionnaires used for data collection by KNHANES included scale-based assessments for health-related quality of life (QOL). Health-related QOL was measured using the validated Korean version of the 5-item self-administered EuroQOL instrument (EQ-5D). The EQ-5D is a generic questionnaire used to assess the QOL in patients with chronic disease [11]. In previous studies, good correlations between EQ5D-index and SGRQ score [12, 13] and between SGRQ score and CAT score were reported [14]. The EQ-5D has a descriptive system and a visual analogue scale (VAS), and reports patients’ current health status. The descriptive assessment consists of 5 items: mobility, self-care, usual activities, pain/discomfort, and anxiety/depression. Each item can be used to represent profiles of health status or can be converted to a summary index (EQ5D index). The VAS is a measurement scale ranging from 0 (worst health status) to 100 (best health status).

### Definition

Degree of airflow limitation was classified based on value of FEV_1_ (% predicted), and GOLD stage 1, 2, 3, and 4 COPD was defined if FEV_1_ > 80%; 50% < FEV_1_ ≤ 80%; 30% < FEV_1_ ≤ 50%; FEV_1_ ≤ 30%, respectively [1]. Body mass index (BMI) was calculated as kg/m^2^. Vitamin D insufficiency was defined as a 25-OHD concentration of ≤ 15 ng/mL. Estimated glomerular filtration rate (eGFR) was calculated using Cockcroft-Gault equation of [(140 − age) × (body weight)]/(serum Cr × 72), with a factor of 0.85 applied for women [14]. Chronic kidney disease was defined when patients reported history of kidney disease or an eGFR of <60 ml/min.

### Statistical analysis

Data were analyzed via complex-sample analysis procedures using SAS version 9.3 (SAS Institute Inc., Cary, NC). We adjusted the analysis for the complex sample design of the survey using stratification, sampling weight variables, and clustering variables for prevalence production. In order to compare characteristics of each subgroup, general linear regression was used for continuous variables and logistic regression was used for categorical variables. To determine the association between lung function and values of biomarkers such as vitamin D and PTH in COPD patients, general linear regressions was conducted with age, sex, height, body mass index (BMI), current smoking status, and kidney disease as variables. With respect to the association between QOL and the values of vitamin D and PTH in COPD patients, adjustment were made for age, sex, BMI, current smoking status, COPD severity, diabetes, hypertension, and kidney disease. Comparisons of QOL according to quartile of biomarker levels were analyzed using analysis of covariance adjusted by same variables. A *P*-value < 0.05 was used to indicate statistical significance.

## Results

A total of 1302 patients met the definition of COPD in data of KNHANES. Among them, 43.5% were GOLD 1 (FEV_1_ ≥ 80%), 51.4% were GOLD 2 (50% ≤ FEV_1_ < 80%), 4.4% were GOLD 3 (30% ≤ FEV_1_ < 50%), and 0.7% were GOLD 4 (FEV_1_ < 30%) group. The mean age of the COPD population was 64.7 (± 0.7) years, 72.5% were males, and 34.1% were current smokers. The prevalence of vitamin D insufficiency (≤15 ng/mL) was 26.9% in COPD patients and 32.4% in non-COPD patients. The participants’ demographic and clinical characteristics of COPD patients as compared with those of the non-COPD control group are summarized in Table 1. Values of both 25-OHD and PTH were significantly higher in COPD group than in the control groups. EQ5D-index and EQ5D-VAS scores were lower in the COPD group (Table 1).

**Table 1:** Baseline characteristics of study population.

		Non-COPD	COPD				*P*	
			Total COPD	COPD 1	COPD 2	COPD 3–4	Non-COPD vs. COPD	Between COPD
Number		8967	1302	567	669	66		
Age		54.3 ± 0.2	64.7 ± 0.4	66.4 ± 0.7	63.1 ± 0.6	65.3 ± 1.7	<0.001	0.001
Male sex		44.5 ± 0.6	72.5 ± 1.8	72.5 ± 2.5	72.2 ± 2.4	79.3 ± 6.3	<0.001	0.821
Height, cm		161.3 ± 0.1	163.0 ± 0.4	163.0 ± 0.5	163.1 ± 0.4	163.5 ± 1.6	<0.001	0.768
Weight, kg		63.7 ± 0.2	62.8 ± 0.4	62.6 ± 0.7	63.1 ± 0.5	60.7 ± 1.7	0.04	<0.001
BMI, kg/cm^2^		24.4 ± 0.04	23.6 ± 0.1	23.5 ± 0.2	23.7 ± 0.2	22.7 ± 0.7	<0.001	<0.001
Smoking							<0.001	0.929
	Current smoking	21.5 ± 0.6	34.1 ± 1.8	32.9 ± 2.5	35.4 ± 2.4	35.0 ± 8.5	0.42	0.438
	Smoking amount, PY	7.7 ± 0.3	18.4 ± 1.3	19.1 ± 1.5	15.7 ± 2.8	23.5 ± 7.6	<0.001	0.640
Underlying ds								
	DM	13.0 ± 0.5	19.7 ± 1.5	17.3 ± 2.2	21.2 ± 2.1	18.1 ± 6.2	<0.001	<0.001
	HTN	39.6 ± 0.7	53.9 ± 1.8	54.7 ± 2.7	51.8 ± 2.6	49.6 ± 8.0	<0.001	0.895
	HL	51.5 ± 1.1	59.4 ± 2.3	58.4 ± 3.1	59.4 ± 3.1	46.9 ± 10.0	<0.001	0.269
	CKD	10.0 ± 0.4	28.7 ± 1.9	33.2 ± 3.1	24.1 ± 2.2	25.0 ± 6.1	<0.001	0.985
PFT								
	FVC, L	3.52 ± 0.01	3.54 ± 0.03	3.88 ± 0.06	3.36 ± 0.04	2.68 ± 0.12	0.548	<0.001
	FVC, %predict	93.1 ± 0.2	90.1 ± 0.5	99.3 ± 0.7	84.5 ± 0.5	66.8 ± 2.0	<0.001	<0.001
	FEV_1_, L	2.81 ± 0.01	2.24 ± 0.03	2.56 ± 0.04	2.08 ± 0.03	1.24 ± 0.05	<0.001	<0.001
	FEV_1_, %predict	94.4 ± 0.2	77.6 ± 0.5	90.4 ± 0.5	69.5 ± 0.4	42.5 ± 1.0	<0.001	<0.001
	FEV_1_/FVC, %	79.9 ± 0.1	63.0 ± 0.3	66.1 ± 0.2	61.9 ± 0.4	47.9 ± 1.5	<0.001	<0.001
Laboratory findings								
	WBC, /μL	6059 ± 25	6589 ± 80	6487 ± 127	6654 ± 103	6723 ± 273	<0.001	0.665
	Hb, g/dL	14.0 ± 0.0	14.5 ± 0.1	14.3 ± 0.1	14.6 ± 0.1	14.5 ± 0.2	<0.001	0.276
	PLT (x 10^3^), /μL	253.9 ± 0.9	244.1 ± 2.2	241.7 ± 2.9	244.4 ± 3.3	270.2 ± 11.3	<0.001	0.096
	BUN, mg/dL	14.9 ± 0.1	15.8 ± 0.2	16.0 ± 0.3	15.7 ± 0.3	15.0 ± 1.0	<0.001	<0.001
	Cr, mg/dL	0.83 ± 0.00	0.91 ± 0.01	0.92 ± 0.01	0.90 ± 0.01	0.92 ± 0.03	<0.001	0.183
	**Vitamin D, ng/mL**	**18.6 ± 0.2**	**19.5 ± 0.3**	**19.6 ± 0.4**	**19.0 ± 0.4**	**20.9 ± 1.3**	**0.001**	**0.002**
	**PTH, pg/mL**	**67.0 ± 0.6**	**70.2 ± 1.2**	**67.7 ± 1.6**	**71.6 ± 1.4**	**78.3 ± 5.9**	**0.005**	**0.032**
Quality of Life								
	EQ5D-index	0.94 ± 0.01	0.91 ± 0.01	0.90 ± 0.01	0.92 ± 0.01	0.88 ± 0.03	<0.001	0.29
	EQ5D-VAS	78.2 ± 0.9	71.7 ± 0.7	71.7 ± 1.1	73.8 ± 2.0	67.0 ± 2.9	<0.001	0.30

Data were respresented as mean ± standard error, or frequency (%)

Abbreviation: BMI, body mass index; PY, pack-year; DM, diabetes; HTN, hypertension; HL, hyperlipidemia; CKD, chronic kidney ds; FVC, forced vital capacity; FEV_1_, forced expiratory volume in 1 second;; WBC, white blood cell; Hb, hemoglobin; PLT, platelet; BUN, blood urea nitrogen; Cr, creatinine; PTH, parathyroid hormone; EQ5D, Euro quality of life instrument; VAS, visual analogue scale

### Vitamin D, PTH, and lung function

**Fig 1:**
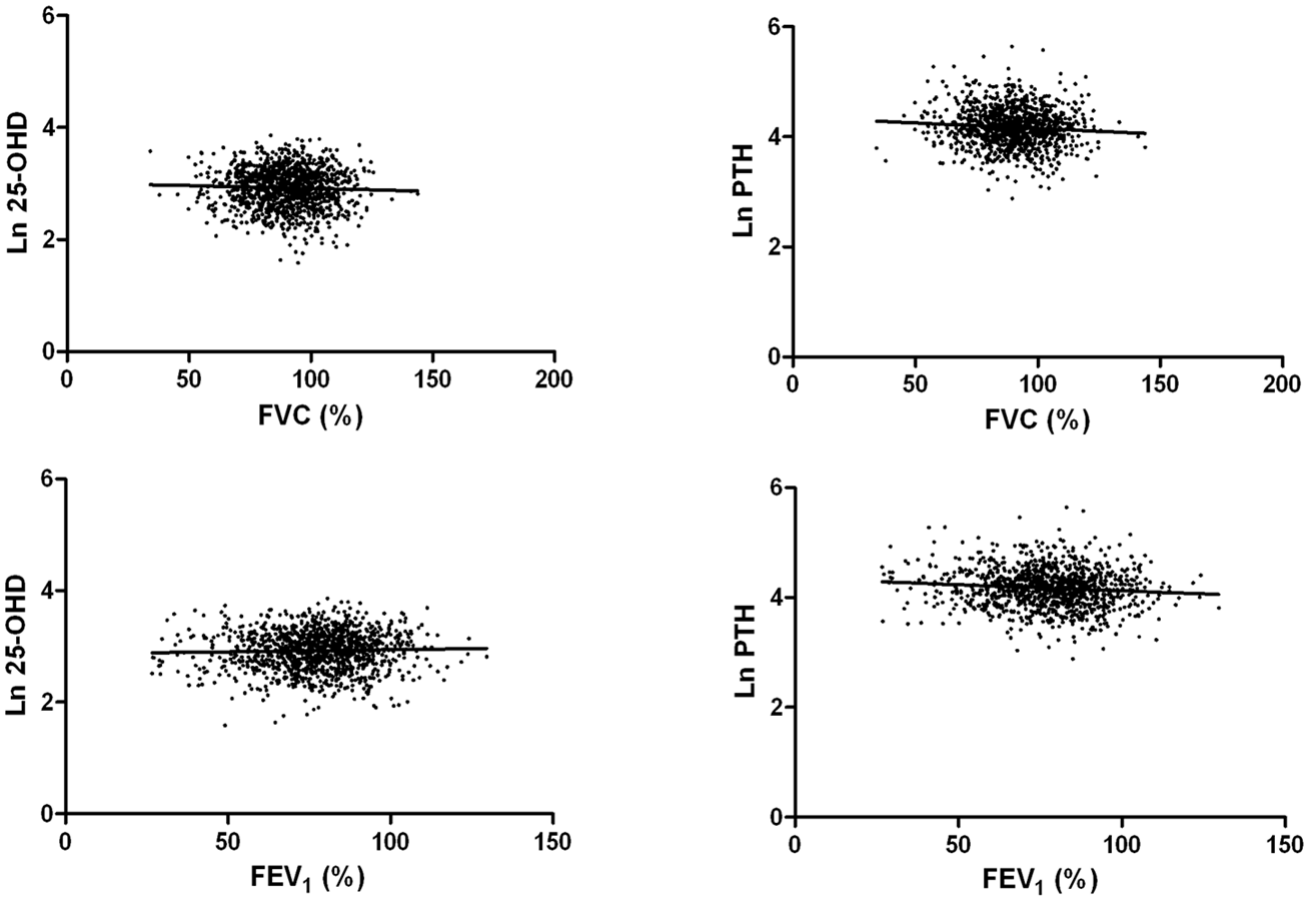
Relationship between 25-hydroxyvitamin D, parathyroid hormone levels and lung function. Log 25-OHD: ß for FVC (%), - 0.04, *P* = 0.16; ß for FEV_1_ (%), 0.03, *P* = 0.24. Log PTH: ß for FVC (%), - 0.08, *P* = 0.01; ß for FEV_1_ (%), - 0.10, *P* = 0.001.

Since values of 25-OHD and PTH were not normally distributed, log transformations of these values were performed to evaluate their association with pulmonary function or quality of life. In COPD patients, the association between 25-OHD levels and FVC (% predicted) or FEV_1_ (% predicted) was not significant in univariable analysis (Fig 1). However, there was a negative correlation between PTH level and both FVC (% predicted) and FEV_1_ (% predicted) (Fig 1), although PTH levels had a significant association with vitamin D levels (ß = 0.18; *P* < 0.001). Multivariable analysis of the relationship between lung function variables and values of vitamin D or PTH were performed, with each analysis adjusted for age, sex, height, BMI, current smoking status, and kidney disease. Vitamin D levels were not significantly associated with either FVC (% predicted) or FEV_1_ (% predicted) in multivariable analysis (Table 2). In contrast, PTH values were independently correlated with decreased FVC (% predicted) (ß = - 0.07; *P* = 0.02) and FEV_1_ (% predicted) (ß = - 0.11; *P* < 0.001) (Table 2).

**Table 2:** Multivariable analysis for association between values of vitamin D, parathyroid hormone, and pulmonary function.

N = 1302		Log 25-OHD		Log PTH	
		ß (95% CI)	*P*	ß (95% CI)	*P*
Pulmonary function					
	FVC (%)	- 0.02 (- 0.07, 0.04)	0.52	- 0.07 (- 0.13, - 0.01)	0.02
	FEV_1_ (%)	0.03 (- 0.03, 0.09)	0.30	- 0.11 (- 0.17, - 0.05)	<0.001

Adjusted by age, sex, height, body mass index, and current smoking status

Abbreviation: FVC, forced vital capacity; FEV_1_, forced expiratory volume in 1 second; 25-OHD, 25-hydroxyvitamin D; PTH, parathyroid hormone

Since age, sex, BMI, smoking status, and underlying disease were different between non-COPD and COPD groups, the analysis of vitamin D values was adjusted by age, sex, BMI, current smoking status, and chronic kidney disease. Differences of adjusted log-vitamin D levels were neither apparent between non-COPD and COPD group (*P* = 0.06), nor among GOLD 1, 2, and 3–4 COPD patients (*P* = 0.40). Between non-COPD and COPD groups, difference of adjusted log-PTH levels also did not reach statistical significance (*P* = 0.08); however, among COPD patients, there was significant increase of adjusted log-PTH level with severity of airflow limitation (*P* = 0.03; Table 3).

**Table 3:** Adjusted values and mean difference of vitamin D and parathyroid hormone levels relative to the presence of COPD and COPD severity.

		Log 25-OHD			Log PTH		
		Mean value (± SE)	Mean difference (± SE)	*P*	Mean value (± SE)	Mean difference (± SE)	*P*
Non-COPD vs. COPD				0.06			0.08
	Non-COPD (N = 8967)	2.87 (± 0.01)	―		4.17 (± 0.01)	―	
	COPD (N = 1302)	2.83 (± 0.02)	- 0.04 (± 0.02)		4.21 (± 0.02)	0.04 (± 0.02)	
Among COPD				0.40			0.03
	GOLD 1 (N = 567)	2.85 (± 0.03)	―		4.19 (± 0.03)	―	
	GOLD 2 (N = 669)	2.89 (± 0.03)	0.04 (± 0.04)		4.21 (± 0.03)	0.03 (± 0.04)	
	GOLD 3–4 (N = 66)	2.82 (± 0.07)	- 0.03 (± 0.07)		4.38 (± 0.07)	0.19 (± 0.07)	

Adjusted by age, sex, body mass index, current smoking status, and chronic kidney disease

Abbreviation: COPD, chronic obstructive pulmonary disease; GOLD, global initiative for chronic obstructive lung disease; 25-OHD, 25-hydroxyvitamin D; PTH, parathyroid hormone

### Vitamin D, PTH, and quality of life

**Fig 2:**
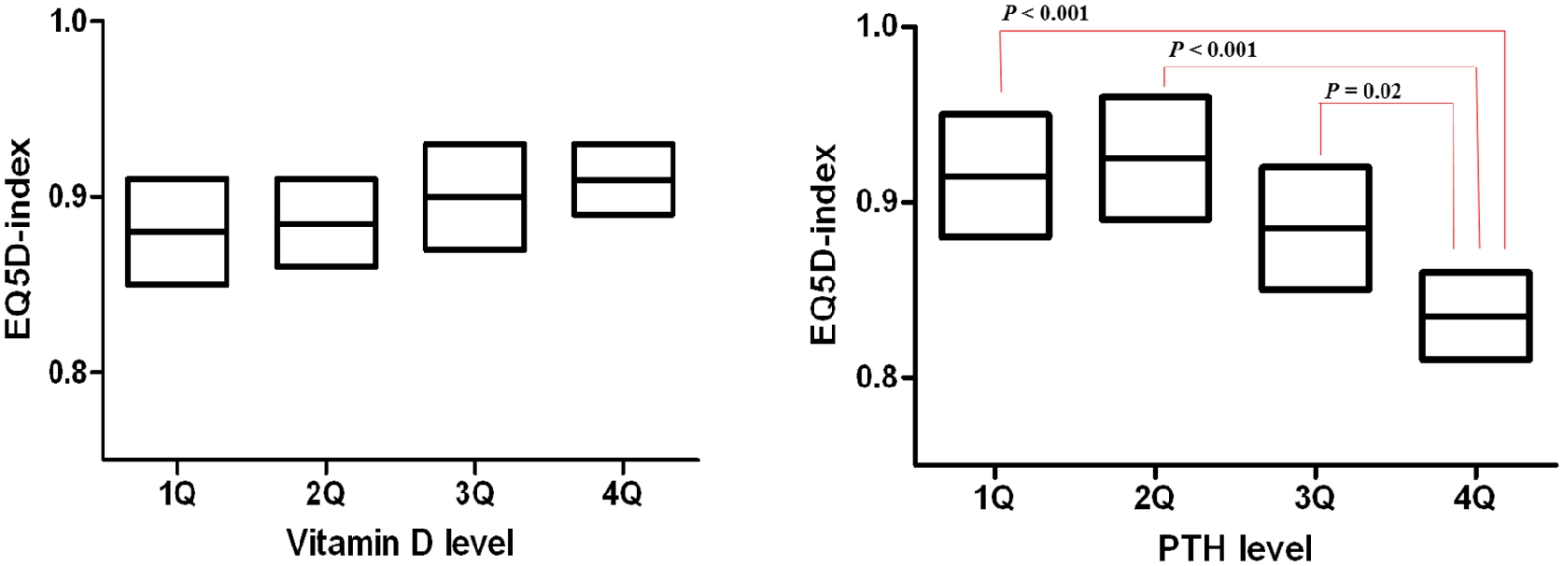
Comparison of adjusted value of quality of life according to quartile of vitamin D and PTH levels. Values were adjusted by age, sex, BMI, current smoking status, kidney disease, GOLD stage, and quartile of vitamin D or PTH level.

Vitamin D level was not correlated with EQ5D-index or EQ5D-VAS score, but PTH level was correlated with EQ5D-index (ß = - 0.08; *P* = 0.01) (Table 4). Multivariable analysis of the relationship between quality of life and values of vitamin D or PTH were performed, with each analysis adjusted for age, sex, BMI, current smoking status, COPD severity, diabetes, hypertension, and chronic kidney disease. Value of vitamin D had no significant relationship with either EQ5D-index or EQ5D-VAS score; however PTH level was significantly associated with EQ5D-index (ß = - 0.06; *P* = 0.04) in multivariable analysis (Table 4). Correlation of PTH with EQ5D-VAS score was not significant (*P* = 0.45). EQ5D-index adjusted by same variables was compared according to quartile of vitamin D and PTH. Quartile (Q) cutoff values were Q1 < 13.20, Q2 13.21–16.80, Q3 16.81–21.20, Q4 ≥ 21.21 for 25-OHD; and Q1 < 50.70, Q2 50.71–63.70, Q3 63.71–80.30, Q4 ≥ 80.31 for PTH. Adjusted EQ5D-index was not different between quartiles of vitamin D (*P* = 0.54), but significantly different between quartiles of PTH (*P* < 0.001). Decline of QOL was apparent in fourth quartile (Fig 2). There was no statistical interaction between airflow limitation and PTH level for EQ5D-index (*P* = 0.23) and EQ5D-VAS score (*P* = 0.54).

**Table 4:** Association between values of vitamin D and parathyroid hormone, and quality of life.

N = 1302		Univariable analysis				Multivariable analysis			
		Log 25-OHD		Log PTH		Log 25-OHD		Log PTH	
		ß (95% CI)	*P*	ß (95% CI)	*P*	ß (95% CI)	*P*	ß (95% CI)	*P*
Quality of life									
	EQ5D-index	0.03 (- 0.02, 0.09)	0.25	- 0.08 (- 0.14, - 0.02)	0.01	0.02 (- 0.04, 0.07)	0.59	- 0.06 (- 0.12, - 0.01)	0.04
	EQ5D-VAS	0.01 (- 0.05, 0.06)	0.81	- 0.04 (- 0.10, 0.02)	0.19	- 0.01 (- 0.06, 0.05)	0.81	- 0.02 (- 0.08, 0.04)	0.45

Adjusted by age, sex, body mass index, current smoking status, COPD severity, diabetes, hypertension, and chronic kidney disease

Abbreviation: EQ5D, Euro quality of life instrument; VAS, visual analogue scale: 25-OHD, 25-hydroxyvitamin D: PTH, parathyroid hormone

## Discussion

In this study, PTH levels were independently associated with FVC (% predicted), FEV_1_ (% predicted), and quality of life in COPD patients. Vitamin D levels were neither associated with lung function nor with quality of life.

In previous studies, vitamin D was regarded as a possible biomarker for COPD. Many recent studies evaluating the role of vitamin D in COPD patients produced controversial results. Peter et al. showed a relationship between serum vitamin D and lung function in national survey data [2]. Afzal et al. reported that lower vitamin D levels are associated with faster FEV_1_ decline rate, higher prevalence of COPD, and higher risk of COPD development [3]. However, another prospective study by Skaaby et al. showed that although lower vitamin D levels correlated with higher COPD prevalence, vitamin D deficiency was not significantly associated with COPD development [4]. Furthermore, Puhan et al. reported no significant correlation between vitamin D deficiency and COPD exacerbation or mortality [7]. Additionally, Jackson et al. observed no difference of vitamin D levels between non-COPD and COPD groups, but described the elevation of PTH level in COPD patients [15]. In our national survey data, 25-OHD level was lower in the control population than COPD patients, and 25-OHD level was not significantly associated with both lung function and quality of life.

One possible explanation for these discrepancies on the role of vitamin D in COPD patients is the complex interaction between vitamin D and PTH. In the human body, vitamin D level is controlled by various factors including PTH, in order to maintain calcium homeostasis [16]. Because most of these studies did not measure the level of PTH which interacts with vitamin D, the effect of PTH on COPD could not be conclusively evaluated. Until now, we focused on the role of vitamin D in COPD without realizing the impact of PTH in COPD patients. Surprisingly our study suggests that not vitamin D, but PTH could be the key biomarker in COPD; however, owing to their interaction, vitamin D was identified as the indicator in many former studies. Previous report by Jackson et al. which showed elevation of adjusted PTH level in COPD patients also supports our findings [15]. The major role of PTH is regulation of bone health and mineral homeostasis, but excess PTH may have possible effects beyond the regulation of calcium homeostasis. Though we could not evaluate the mechanism of poorer pulmonary function in PTH elevation, PTH might be a more effective biomarker than vitamin D for COPD patients, especially in regions with a high prevalence of vitamin D insufficiency. Furthermore, value of PTH could be independent predictor for quality of life regardless of degree of airflow limitation. In current GOLD guideline, staging of COPD is classified by both degree of airflow limitation and symptom score, and the importance of symptom score is emphasized by higher mortality rate [17]. Levels of PTH, as well as vitamin D, are known to have reverse J-shaped association with mortality, and both elevated and decreased levels of PTH increase all-cause mortality in the general population [18, 19]. Although we did not observe the association between lower PTH levels with poor pulmonary function or quality of life, we did observe the association between higher PTH levels and decreased pulmonary function and quality of life, both of which can increase mortality in COPD patients. Further studies are needed to determine whether PTH affects lung tissue or lung disease causes secondary hyperparathyroidism.

Since the present study describes the novel relationship between PTH levels and COPD severity, the following limitations of the study are noted in an effort to aid in the interpretation of our results. First, we did not evaluate post-bronchodilator FEV_1_ and FVC, which are commonly used as lung function parameters in COPD patients, as this study was part of the national health survey. Additionally we could not obtain data on diffusing capacity for carbon monoxide (DLCO), emphysema status, coexistence of bronchiectasis, or other variables reflecting functional status such as the 6-minute walk distance, CAT score, SGRQ score. Hence, we used EuroQOL as the QOL index, which is a generic, but not a disease-specific questionnaire. Second, we could not evaluate serum calcium levels, which prevent calculation of prevalence of primary hyperparathyroidism. Third, we could not evaluate the seasonal variation of vitamin D and PTH, because limited access to the time of sampling owing to ethical concern for personal information. Fourth, we could not completely differentiate COPD from asthma, because KNAHNES included the self-reported questionnaires for asthma, but most patients did not perceive the difference of these diseases. Therefore, there could be contamination of longstanding, smoking-related asthma or overlap syndrome in our COPD population. As we could not identify the possible mechanism for these results, further detailed, larger trials to evaluate the role of PTH in COPD prevalence, exacerbation, lung function decline rate, functional status, and mortality are needed to confirm our findings.

In conclusion, elevation of PTH, unlike vitamin D, is independently associated with poorer lung function and quality of life. PTH may be a better biomarker for COPD patients, especially in regions with a high prevalence of vitamin D insufficiency.
